# Variable Stiffness Conductive Composites by 4D Printing Dual Materials Alternately

**DOI:** 10.3390/mi13081343

**Published:** 2022-08-19

**Authors:** Fei Long, Gaojie Xu, Jing Wang, Yong Ren, Yuchuan Cheng

**Affiliations:** 1 Zhejiang Key Laboratory of Additive Manufacturing Materials, Ningbo Institute of Materials Technology and Engineering, Chinese Academy of Sciences, Ningbo 315201, China; 2 Department of Mechanical, Materials and Manufacturing Engineering, University of Nottingham Ningbo China, Ningbo 315100, China; 3 Research Group for Fluids and Thermal Engineering, University of Nottingham Ningbo China, Ningbo 315100, China; 4 Department of Electrical and Electronic Engineering, University of Nottingham Ningbo China, Ningbo 315100, China; 5 Key Laboratory of Carbonaceous Wastes Processing and Process Intensification Research of Zhejiang Province, University of Nottingham Ningbo China, Ningbo 315100, China; 6 Center of Materials Science and Optoelectronics Engineering, University of Chinese Academy of Sciences, Beijing 100049, China

**Keywords:** liquid metal, variable stiffness, 4D printing, thermal response, phase change

## Abstract

Materials that can be designed with programmable properties and which change in response to external stimuli are of great importance in numerous fields of soft actuators, involving robotics, drug delivery and aerospace applications. In order to improve the interaction of human and robots, materials with variable stiffness are introduced to develop their compliance. A variable stiffness composite has been investigated in this paper, which is composed of liquid metals (LMs) and silicone elastomers. The phase changing materials (LMs) have been encapsulated into silicone elastomer by printing the dual materials alternately with three-dimensional direct ink writing. Such composites enable the control over their own stiffness between soft and rigid states through LM effective phase transition. The tested splines demonstrated that the stiffness changes approximately exceeded 1900%, and the storage modulus is 4.75 MPa and 0.2 MPa when LM is rigid and soft, respectively. In the process of heating up, the stretching strain can be enlarged by at least three times, but the load capacity is weakened. At a high temperature, the resistance of the conductive composites changes with the deformation degree, which is expected to be applied in the field of soft sensing actuators.

## 1. Introduction

In contrast to the uniaxial or torsional displacements of traditional actuators, soft elastomers can be programmed to undergo changes throughout the whole structure with the significant advantages in low density, high mechanical flexibility and multidimensional movement. As an aspect that has received a lot of attention surrounding soft actuators recently, tunable stiffness refers to the ability of materials or systems to transform between a compliant and rigid load-bearing state after applying external stimulus. Until now, such controllable stiffness materials have been applied in a wide range of fields, such as more efficient catheters and endoscopes to perform non-invasive procedures [1] in medicine, adaptive wings that improve aircraft performance [2] in aeronautics and building materials that lower the damages from wind and earthquakes [3] in architecture. To avoid time and material consuming with the tedious fabrication process, a suitable approach with additive manufacturing (AM) is required for effective fabrication of 3D structure with complex topographical feature. Taking a particular instance, various 3D printing techniques have been applied for designing versatile microfluidic systems [4] to detect different analytes [5] and different clinically relevant diseases [6].

The additive manufacturing technique, also known as three-dimensional printing (3D printing), can enable one-step patterning of multi-materials, such as plastics [7], metals [8], ceramics [9] and woods [10]. Traditional processing techniques such as milling, molding and engraving result in high ambient temperatures, which have been a hindrance to pattern metal and polymeric materials directly. The conventional 3D printing objects are considered as statistic, whose dynamic evolutions were restricted in potential applications. Recently, the significant efforts on 3D printing have yielded four-dimensional (4D) printing structures that the structure or properties change over time; in other words, it is an updated version of 3D printing technologies with stimuli-materials involving heat [11], light [12], magnetic field [13], electrical field [14], etc. Until now, 4D printing has attached worldwide attention from micro- to macroscale as a result of various functional applications in soft robotics, flexible electronics, and biomedicine [15].

The materials with significant variable stiffness can be primarily classified into three groups that are shape memory polymers (SMP), smart materials and low melting point materials. Shape memory materials can experience the irreversible transitions from metastable to globally stable states [16] or response to environmental conditions to undergo reversible changes (such as LCEs [17]). The phase transition of major shape memory materials operates through temperature change, in detail that SMPs must be heated indirectly using external heaters [18]. For smart materials (for example, electrorheological fluids (ERF) [19] and magnetorheological fluids (MRF)) [20], the principle of phase transitions depends on the order of the inner nanometer or micrometer to change own viscosity. Low melting point materials represented by metal alloys [21] can be heated directly due to the thermal conductivity, and its heating rate is almost 100 times of SMPs with 18 W/mK [22]. Since both smart fluids and low melting point materials will exist as flowing state in a compliant state, they must be encapsulated to perform stiffness-changing functions without the loss of fluids.

As one of the low melting point materials, liquid metals (LMs) exhibit the properties of traditional rigid metal in a solid state, while it can be dispensed, stretched and deformed easily after melting. With the inherent soft state, LMs are suitable for applications in the devices where the desired materials need to endure varying degrees of stress, such as soft actuators and soft robotics. In addition, the features in high electrical and thermal conductivity can be applied in cases where design stretchable circuits and strain sensors [23], such as heat dissipation [24]. However, the surface tension of LMs is high with approximately 700 mN/m [25], that is poor wettability with solid substrates, resulting in the leakage or spillage problems when utilizing devices [26]. Cheng et al. [27] reported that the oxidized LMs have ability to reduce surface tension and improve wettability, but its lower thermal conductivity with around 1 Wm^−1^ K^−1^ will deteriorate severely. Until now, to balance wettability and thermal performance, Cu particles have been mixed by various shapes and sizes [26]. Due to LMs fluidity in the liquid state and inherent brittleness in the stiff state, it is difficult to pattern in the solid form for further processing [28]. It is possible to introduce direct ink printing (DIW) that is a common approach in the AM field in spite of existing challenges to print LM directly [29], so the precise control of distance between nozzle and surface enables the direct printing of non-spherical shapes by shearing metal from the nozzle. As the printed structures maintain a liquid state at room temperature, further encapsulations are required for most applications.

In this paper, a controllable stiffness composite has been proposed, which consisted of LM as functional materials and silicone elastomer as the encapsulation layer, respectively. Here, to explore the absolute stiffness and hardness, three types of silicone have been selected, including Ecoflex00-30, PDMS Sylgard 184 and SE 1700. Additionally, different mixing ratios of silicone have been adjusted to satisfy the necessary rheological conditions for DIW. Through printing dual materials alternately, simple splines, hollow flower patterns and Poisson structures can be fabricated. The stiffness change of LM composites can be controlled by the thermal response of LMs, which results in large changes in stiffness after LM melting completely, while the silicone guarantees that the melted LMs retain the pre-molten shape. The DIW of LMs has broken through the difficulty of filling approach and can achieve a larger volume content of LM composites that were promoted a wider range of stiffness change. At a temperature above the melting point, the addition of LM will greatly increase the tensile deformation capacity, but the load-carrying ability will be weakened to a certain extent. Owing to the electrical conductivity of LMs, their resistance can change during stretching and the recyclability has certain advantages compared to previous studies, which is expected to be applied into soft sensing actuators.

## 2. Experimental

### 2.1. Dual Phase Direct Write Printing

**Figure 1:**
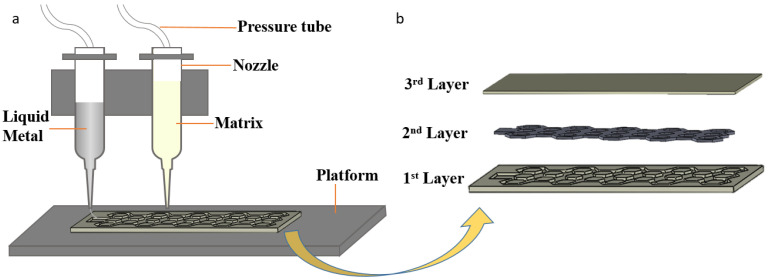
(**a**) The schematic diagram of dual phase direct write printing. (**b**) The design structure in a ‘sandwich’ structure.

A challenge for LM elastomers is that filling LM into the cured silicone mold is difficult at the room temperature due to the high surface tension of LM. A multi-material alternate printing approach, previously used for water-soluble support in 3D bioprinting systems has been proposed to stabilize the surface contact between materials. Bodaghi et al. [30] has fabricated dual-material lattice-based meta-structures by fused deposition modelling technology. In this study, the progress of dual phase direct write printing (Figure 1a) has been divided into three layers as a ‘sandwich’ structure, shown as in Figure 1b. In the DIW printing process, the relevant typical parameters can refer to the 3D printing of stretchable elastomers by Zhou et al. [31]. With a suitable pressure P = 0.1 MPa, the slurries are extruded from the narrow nozzle with a diameter D of 450 mm at a speed c which is pressure dependent. Since the extruded slurries will experience die-swelling [32], the diameter of the filament can be defined as αD, in which α is die-swelling ratio. Meanwhile, the deposited slurries are extruded from the moving nozzle at a speed V of 8 mm/s and a height H of 0.05 mm which is the distance between the printer layer and the nozzle. As the surface tension between the LM in solid state and the silicone elastomer in semi-solid state is relatively small [33,34], cooling LMs to solid can alleviate such tough problems. In detail, after completing the first layer, the carrier platform needs to be cooled down to −10 °C for printing the second layer, which ensures that LMs can be cured quickly after printing to contact with the first layer in the solid state. Finally, the third layer is printed to encapsulate the LMs. This one-step additive manufacturing technology can complete complex structures in a short time, effectively reducing preparation time and saving costs. The process of extruding the slurries from the narrow nozzle can be regarded as preparing the tiny droplets under the microfluidic control, so the rheological parameters are extremely significant.

### 2.2. Materials

The liquid metal applied in this research is composed of 75.5% gallium (Ga) and 24.5% indium (In), purchased from Jintai Alloy Corporation, Guangdong, China. The selected LMs start to melt from 20 °C to 40 °C approximately, which has been verified by DSC measurements, as shown in Figure S1. At room temperature, LMs remain liquid state of high surface tension with around 0.624 N/m and low viscosity with 0.0024 Pas; the above data were provided by supplier, which are consistent basically with previous study by Koster [35]. Here, there are three types of silicon elastomers selecting in this study, containing EcoflexTM 0030 (Smooth-On, Macungie, PA, USA), PDMS 184 (DOWSILTM, Sylgard, Midland, MI, USA) and PDMS 1700 (DOWSILTM, SE, Midland, MI, USA). Ecoflex 0030 was prepared in a 1:1 base to curing agent weight ratios, while two kinds of PDMS were mixed at 1:10. In order to obtain the printable slurries, Ecofelx 0030 and SE1700 would be mixed at 1:1 in weight, while Sylgard 184 and SE 1700 should be mixed at 1:2. The printable substrates have been mixed with a planetary mixer (VM300SA3, Miantangshinuo Corporation, Jiangsu, China).

### 2.3. Measurements

#### 2.3.1. Thermal Characterization

The experiments on the melting point of LMs were used a DCS 214 (NETZSCH, Selb, German) with high purity alumina ceramic crucible that can withstand 100 °C and bear the corrosion of Ga. The measurement temperature range was from −40 °C to 40 °C, and the heating rate was 10 K/min.

#### 2.3.2. Rheological Characterization

The experiments on rheology were conducted by Discovery HR-20 (TA, New Castle, DE, USA), equipped with a 20 mm parallel plate geometry. To minimize the effect by measuring, all samples were pre-sheared and tested for three times. Additionally, the specific steps have been discussed in further detail in the ESI.

#### 2.3.3. Mechanical Characterization

Mechanical experiments were conducted using Dynamic Mechanical Analyzer DMA Q800 (TA, New Castle, DE, USA) and universal testing machine UTM Roell Z030 (Zwick, Ulm, German). Both instruments have been equipped with the heating function, and the detail settings have been shown in the ESI.

#### 2.3.4. Electrical Characterization

A high-precision LCR digital bridge TH2827c (Tonghui, Changzhou, China) was used to confirm whether the elastomer is conductive. The change of resistance during the stretch progress was monitored, and related electrical data has been synchronized directly to the computer.

## 3. Results and Discussions

### 3.1. Rheological Properties of Matrix

**Figure 2:**
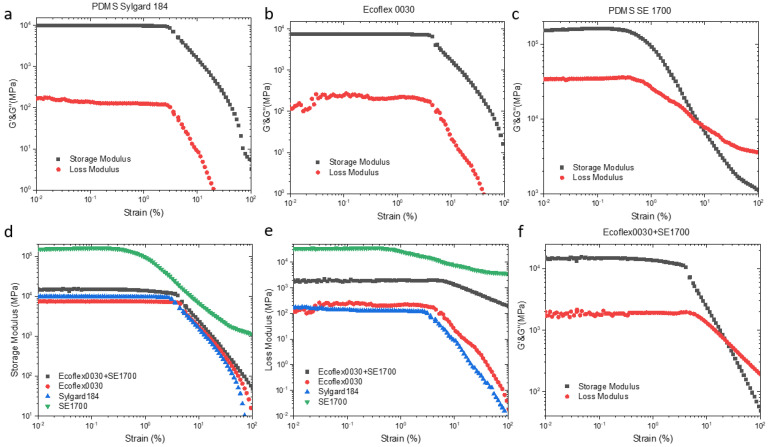
Viscoelastic inversion measurement of (**a**) PDMS Sylgard 184, (**b**) Ecoflex 0030 and (**c**) PDMS SE 1700. (**d**) The storage modulus and (**e**) loss modulus versus strain for silicone elastomer. (**f**) The viscoelastic inversion characteristics of the combination of Ecoflex 0030 and PDMS SE 1700.

In the progress of DIW, the slurries pass through the narrow constriction of a needle which generates high shear force; once extruded, such shear force disappears instantly. Thus, it is required that the printed slurries can be smoothly extruded from the nozzle and perform well self-supporting after extrusion. In other words, the proportioning material should meet the characterization in both shear thinning [36] that viscosity decreases with shear strain, and viscoelastic inversion [37] that the changes of storage modulus and viscoelastic modulus show an intersection with the increase in shear strain. Inspired by Sangchul et al. [38], the above characterization can be obtained by adding other polymers. Here, SE 1700 was mixed with Ecoflex 00-30 and Sylgard 184, respectively, at a ratio of 1:1 and 1:1.5 in weight. Prior to combining, rheological tests have been carried out on these three silicones, all of which occurred shear thinning, but merely SE 1700 has the characteristic of viscoelastic inversion (Figure 2a–c). The plateau value of storage modulus of SE 1700 is two orders of magnitude larger than that of the other two silicone rubbers, while its loss modulus is larger as well (Figure 2d,e). Furthermore, two types of mixed silica gels were also conducted systematic rheological investigations that reflect direct write printability (Figure 2f). The combination of SE 1700 and Sylgard 184 at a weight ratio of 1:1.5 (as shown in Figure S2) has both the properties of shear thinning and viscoelastic inversion. Additionally, the above materials have been mixed in a 1:1 ratio (consistent with Ecoflex 0030), and G’ and G’’ have no intersecting trajectories (Figure S3), that is, they cannot be applied to DIW printing.

**Figure 3:**
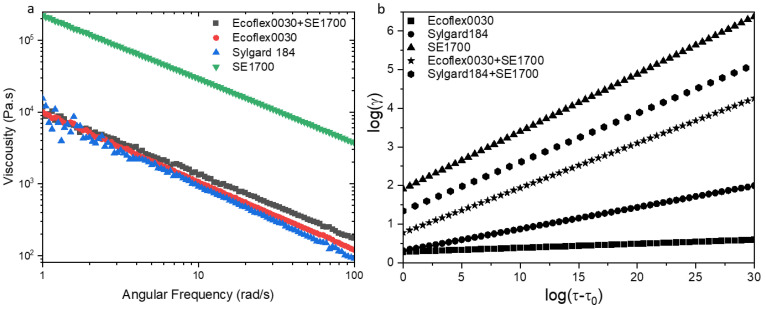
(**a**) Shear thinning of the selected silicone slurries (**b**) The fitting linear function of selected silicone slurries by Herschel and Bulkley model.

The shear thinning behavior can be derived from a power−law variant of Herschel and Bulkley model [39], in detail that if the value of *n* is between 0 and 1 in the fitted linear function relationship based on the Equation (1), the material possesses the characteristics of shear thinning.

(1) $$\tau \;={\;\tau }_{0}+k{\dot{\gamma }}^{n}$$

where τ is shear stress, τ_0_ > 0 is the yield stress, *k* > 0 is the consistency parameter, and *n* > 0 is the power index. Figure 3b can be obtained by fitting a linear function by taking a logarithmic relationship to Equation (1), and all value of *n* below 1. As the increasing of *n*, the phenomenon of shear thinning becomes more obvious. So, the joint of a certain amount of SE 1700 can effectively make the substrate printable.

### 3.2. Mechanical Properties of Matrix

**Figure 4:**
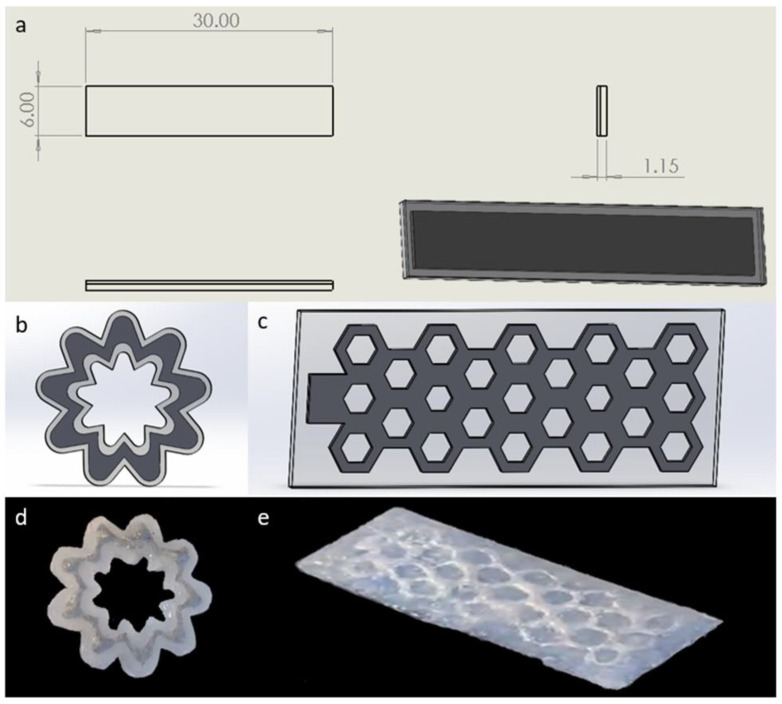
(**a**) The detail engineering diagram of sample splines, (**b**) the design structure, (**c**) the printed real part, (**d**) hollow flower structure and (**e**) Poisson structure.

**Figure 5:**
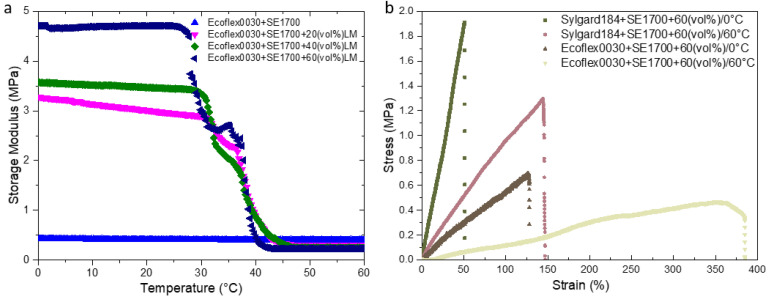
(**a**) The storage modulus with LM composite with the increasing volume fraction of LM. (**b**) The load capacity versus the stretchable strain at low and high temperature.

The stiffness is a vital metric for defining the forces changing that the composites can support. After the printed splines are cured (Figure 4a, and the detail dimensions has been labeled in the engineering drawing), a series of DMA measurements have been established that all the tested LM composites are elastomeric in nature. As shown in Figure S4a, the storage modulus of silicone elastomer mixed with Ecoflex 0030 and SE 1700 is smaller than that of Sylgard 184 but greater than that of SE 1700, which is more conductive to highlight the effect of LM additive. So, the former mixed matrix has been focused on, while the latter analysis can be referred to in the Supporting Information section. Despite that LMs possess fluidity in the liquid state at room temperature, the storage modulus of LM composites increases with LM volume fraction (Figure 5a), compared to the unfilled LM composites with 0.429 ± 0.01 MPa, storage modulus at 60 vol% experienced increasing by a factor of around 11 to 4.71 ± 0.1 MPa. The incorporation of liquid inclusions enables to improve the stiffness of polymer composites to a certain extent, which has been demonstrated [40,41]. In addition, the stiffness is also influenced by the interfacial tension between LM and silicone matrix [42]. Taking example of the composites with 60 vol%, its storage modulus decreases gradually as temperature rises, but a plunge has been occurred when the temperature reaches the melting point T_m_ of LM. Such a dip becomes more pronounced as the LM volume content increases, and the change of storage modulus can be over 200% during the transformation between the rigid and soft state. Moreover, the relationship between deformation strain and load bearing of LM composites has been explored by UTM, as shown in Figure 5b. As a result of the solid-liquid transformation taking place below 60 °C, it can be stretched much more than that keeping at 0 °C, particularly, up to 3.5 times for LM (60 vol%) composites mixed with Ecoflex 0030 and SE 1700. Meanwhile, the corresponding carrying load has been weakened due to the soft state of LM. Similar changes occurred in the LM composites with Sylgard 184 and SE 1700 as the carrier (Figure S5).

Apart from the simple splines, the flower-shaped and Poisson structure have been designed (shown in Figure 4b–e). The modeling and fabrication of relatively complex structures in a short period of time further confirm the high efficiency of additive manufacturing. As the temperature rises, the changes on tensile strength of the above designed structures have been recorded in Movies S1 and S2. In terms of Poisson structures, the connection between bearing capacity and deformation degree at high temperature has been displayed in Figure S6.

### 3.3. Resistance Changes in the Process of Stretching

As the typical types of PCMs, LMs possess good electrical conductivity. The state of LM can be judged by monitoring changes in resistance, thereby determining the softness of composites. In theoretical, the resistance of LM composites can be referred as the standard equation for the wire:

(2) $$R=\frac{\rho L}{A}$$

in which ρ is the electrical resistivity, *L* is the length and *A* is the cross-sectional area of conductor. It can be seen that the resistance varies with the geometry for a certain material. During the stretching process at high temperature, the samples will not only undergo thermal expansion but also length elongation, and the length increases faster than the cross-sectional area.

To characterize the change in resistance, the samples have been clamped on UTM, connecting with LCR digital bridge simultaneously. The relative resistance change has been introduced:

(3) $$\frac{\Delta R}{R_{0}}=\frac{R-R_{0}}{R_{0}}$$

**Figure 6:**
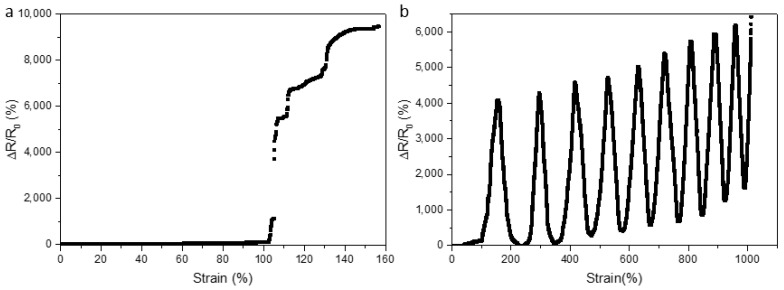
(**a**) At high temperature (60 °C), the relative resistance changes with the stretchable strain, and (**b**) the stretch repeatability over time.

*R* and *R*_0_ are corresponding to the resistance values with and without deformation, respectively. The Δ*R*/*R*_0_ increases with stretching deformation, which proves that the external strain has a certain influence on the relative resistance change. To investigate the sensing performance in terms of tensile strain, its sensitivity can be defined by gauge factor (GF):

(4) $$GF=\frac{\Delta R/R_{0}}{\Delta L/L_{0}}$$

where, *L*_0_ represents the initial size of the splines, and *ΔL* indicates the size change. As shown in Figure 6a, with the good corresponding consistency, the highest strain can reach 9400% approximately, and *GF* value is around 60 at this point. Moreover, the relationship between resistance changes and deformation of LM composites with Sylgard 184 and SE 1700 has been explored (Figure S8a), which can be up to around 120% with the GF value of 1.28 approximately. 

According to the maximum strain obtained from the measurement, the sample of LM composites mixed with Ecoflex 0030 and SE 1700 have been applied to the strain from 0% to 120% at a constant rate of 10 mm/min, and then released until they return to the initial state. The relative resistance change versus time has been achieved in Figure 6b by applying and releasing pressure several times repeatedly, and the spline takes place fracture when the number of cycles is about 10 times. With the increase in strain, Δ*R*/*R*_0_ improves gradually; and after reaching the peak value, Δ*R*/*R*_0_ deceases as a result that the external force is released. The sample can basically return to its original shape when the external force disappears. However, the stretching process will cause a certain degree of permanent loss for no contract of internal LMs fracture due to elastomer stretching, which will make the value of resistance become larger when returning to the origin point. At the tenth stretch, the sample has broken. Moreover, the reproducibility test graph for LM composites with Sylgard 184 and SE 1700 have been demonstrated in the Supporting Information (Figure S8b).

## 4. Conclusions

We have developed a variable stiffness composite that consists of LM and silica gel with different mixing ratios, which can change properties in response to the thermal stimuli. In DIW printing, the process of slurry extrusion can be regarded as material prepared by a microfluidic channel, so the related rheological properties are necessary for the combined slurries. With a certain printability, dual material printing alternately has been applied to fabricate LM composites in one step for the relative complex structures. The samples presented here illustrate the stiffness change of greater than 1900% from a stiff to soft state, while the storage modulus decrease from 4.75 MPa and 0.2 MPa after heating up. Furthermore, by changing the inner structure design or volume fraction between LMs and silicon elastomer, different stiffness values for these two steady states can be achieved. Owing to the electrical conductivity of LMs, the composite exhibits electrical resistance that changes with stretching. However, each stretch will lead to irreversible damage in the elastomer to a certain extent; the spline fracture is generated after about ten repetitions of the tensile test. Overall, this work has demonstrated the LM composites undergo the changes in mechanical and electrical properties under temperature stimuli. With the tuning capability, LM composites are expected to be used in the field of soft sensing actuators, even towards artificial muscle applications after enhancing adhesion.

## Data Availability

The data that support the findings of this study are available from the corresponding author upon reasonable request.

## Supplementary Materials

The following supporting information can be downloaded at: https://www.mdpi.com/article/10.3390/mi13081343/s1; Figure S1: The thermal properties of LM by DSC measurements; Figure S2: a. Shear thinning and b. Viscoelastic inversion measurement of the combination of PDMS Sylgard 184 and PDMS SE1700 with 1:1.5 in weight ratio; Figure S3: a. Shear thinning and b. Viscoelastic inversion measurement of the combination of PDMS Sylgard 184 and PDMS SE1700 with 1:1 in weight ratio; Figure S4: The storage modulus of splines composed by different silicone elastomer a. Comparison with Sylgard 184, SE 1700 and their mixture based on 1:2 in volume fraction. b. Comparison with Ecoflex 0030, SE 1700 and their mixture on the basis of volume fraction with 1:1; Figure S5: Th storage modulus of LM composites that subtracted by Sylgard184 and SE1700 with the LM increasing volume fraction; Figure S6: The load capacity versus the stretchable strain at low and high temperature for the Poisson structure with 60 vol% in LM; Figure S7: The Schematic diagram of real-time monitoring resistance measurements; Figure S8: At high temperature (60 °C), the relative resistance of LM composites (that based on the mixture of Sylgard184 and SE 1700) changes with the stretchable strain, and b. the stretch repeatability over time; Movie S1: Flower; Movie S2: Poisson Structure.
