# Supplementary material for: Variable Stiffness Conductive Composites by 4D Printing Dual Materials Alternately
Source: Micromachines (Basel). 2022 Aug 19;13(8):1343. doi: 10.3390/mi13081343 (PMC9415883; doi:10.3390/mi13081343)
Supplement: Supplementary file 1 [file micromachines-13-01343-s001.zip › micromachines-1858165-supplementary.pdf]

Variable stiffness conductive composites by 4D printing dual materials alternately

Fei Long[1,2,3], Gaojie Xu[1], Jing Wang[4], Yong Ren[2,3,5*], Yuchuan Cheng[1,6*]

1. Zhejiang Key Laboratory of Additive Manufacturing Materials, Ningbo Institute of Materials Technology and Engineering, Chinese Academy of Sciences, Ningbo, 315201, China
2. Department of Mechanical, Materials and Manufacturing Engineering, University of Nottingham Ningbo China, Ningbo, 315100, China
3. Research Group for Fluids and Thermal Engineering, University of Nottingham Ningbo Nottingham, Ningbo, 315100, China
4. Department of Electrical and Electronic Engineering, University of Nottingham Ningbo China, Ningbo, 315100, China
5. Key Laboratory of Carbonaceous Wastes Processing and Process Intensification Research of Zhejiang Province, University of Nottingham Ningbo China, 315100, Ningbo, China
6. Center of Materials Science and Optoelectronics Engineering, University of Chinese Academy of Sciences, Beijing 100049, China

* Corresponding authors:

E-mail: Yuchuan Cheng yccheng@nimte.ac.cn; Yong Ren yong.ren@nottingham.edu.cn

Supporting Information

Section 1 The thermal measurements of LM

Section 2 The rhetorical properties of LM composites

Section 3 The mechanical properties of LM composites

Section 4 The electrical Properties of LM composites

Section 1. The thermal measurements of LM

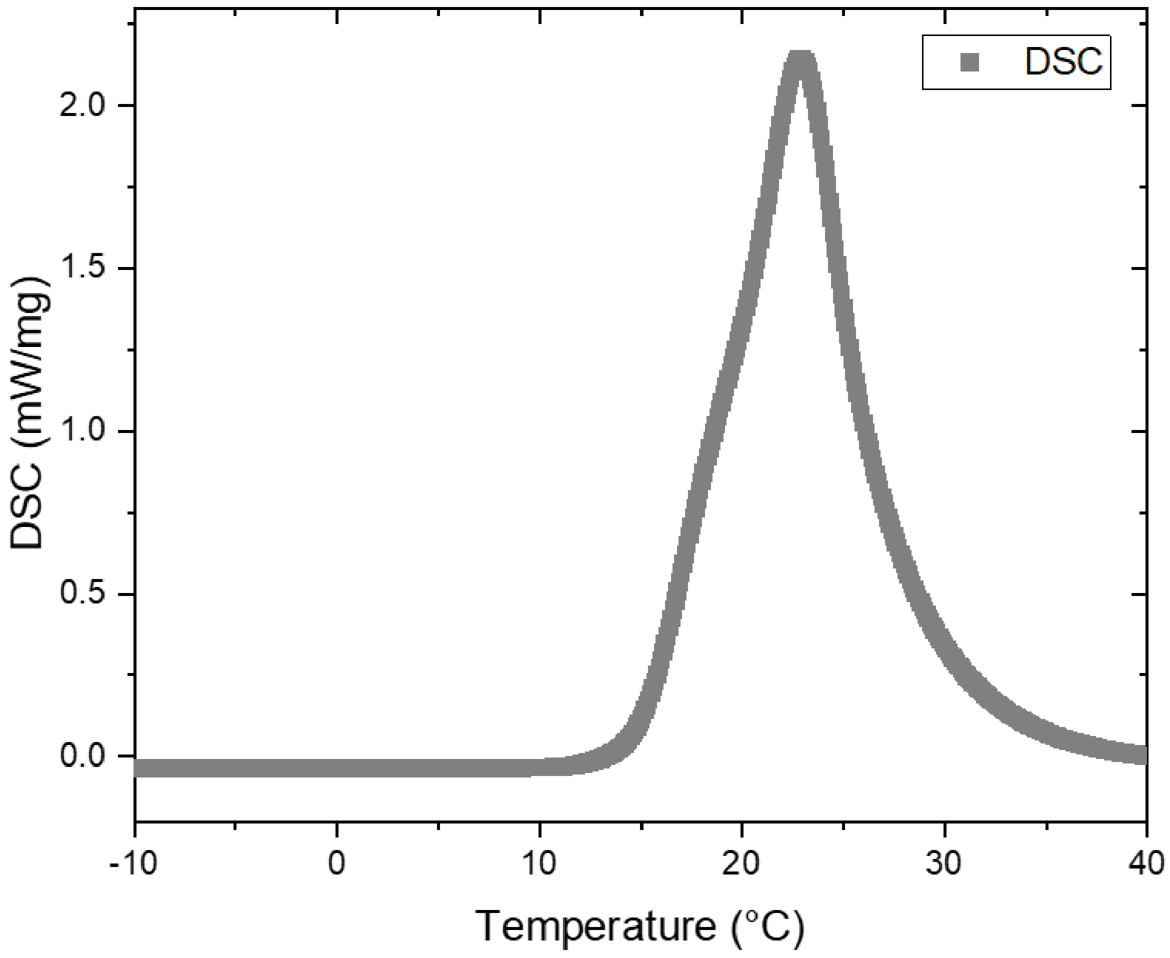

Figure S1 The thermal properties of LM by DSC measurements

The DSC results of LMs reflect that it starts melting from around 15°C, and completely melts till 35°C approximately. The initial melting value is basically similar as that provided by the supplier.

Section 2. The rhetorical properties of LM composites

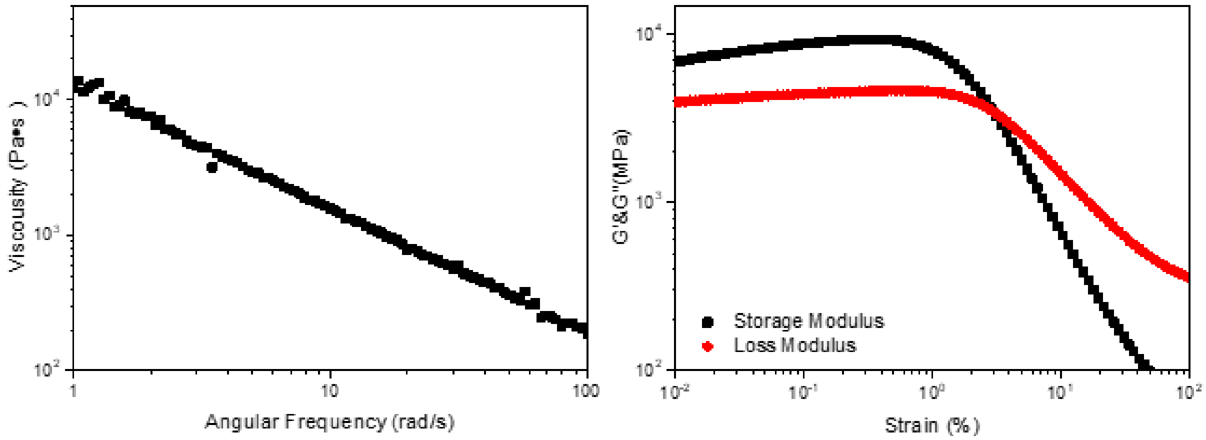

Figure S2 a. Shear thinning and b. Viscoelastic inversion measurement of the combination of PDMS Sylgard 184 and PDMS SE1700 with 1:1.5 in weight ratio.

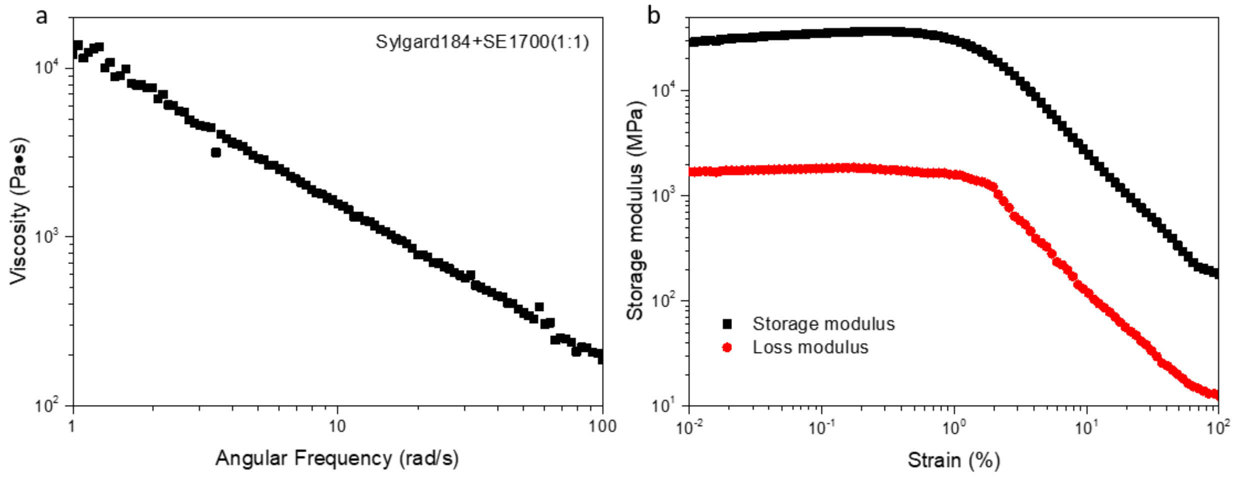

Figure S3 a. Shear thinning and b. Viscoelastic inversion measurement of the combination of PDMS Sylgard 184 and PDMS SE1700 with 1:1 in weight ratio.

All the rheological measurements were conducted using Discovery HR-20 (TA, USA), equipped with a 20mm parallel plate geometry at a gap height of 1mm. In the initial step, the test condition has been set with Frequency Sweep at 0.01% strain from 1.0o 100.0ad/s for the relationship between viscosity and angular frequency. Then, the Oscillatory Amplitude Sweep has been applied to measure the characteristic of viscoelastic inversion at a frequency of 10.0 ad/s from the strain of 10^{-3} to 100%.

Section 3. The Mechanical properties of LM composites

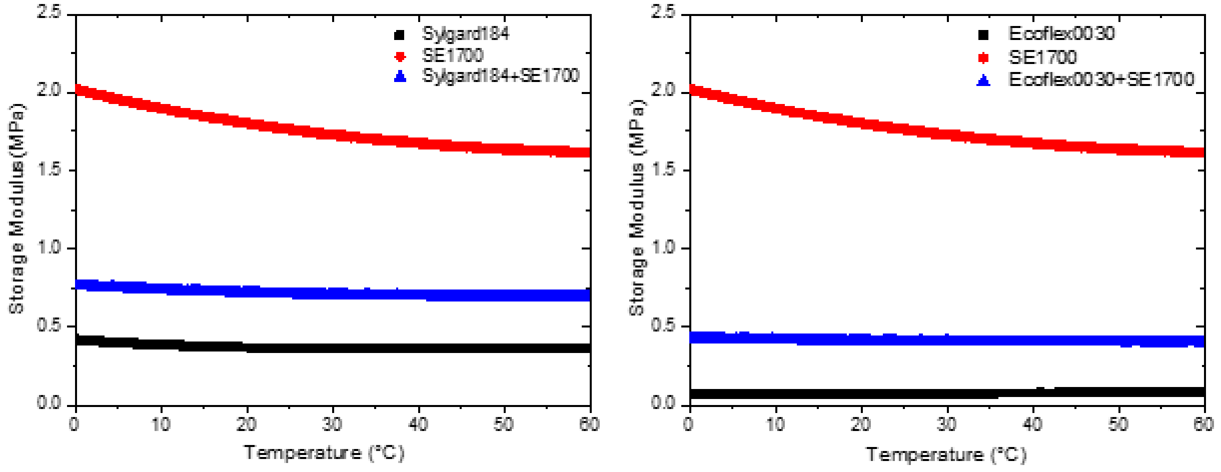

Figure S4 The storage modulus of splines composed by different silicone elastomer a. Comparison with Sylgard 184, SE 1700 and their mixture based on 1:2 in volume fraction. b. Comparison with Ecoflex 0030, SE 1700 and their mixture on the basis of volume fraction with 1:1.

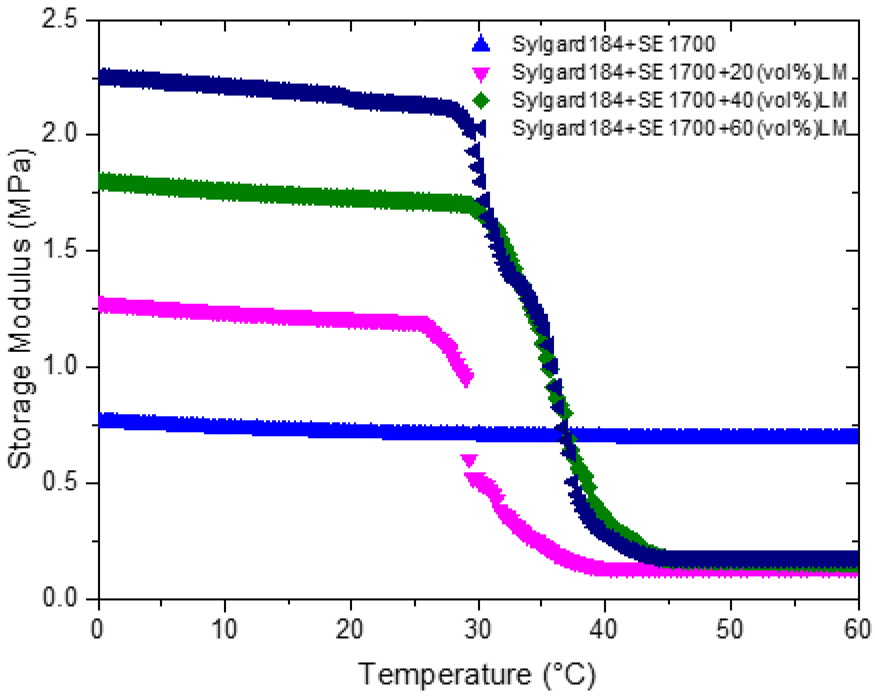

Figure S5. Th storage modulus of LM composites that subtracted by Sylgard184 and SE1700 with the LM increasing volume fraction.

The mechanical properties of LM composites with corresponding transition temperature were conducted by DMA (Q800) of the printed samples with 25mm×5mm×1mm. The environmental condition for implementation is 0.01N preload force and 155% force track at 20.0 µm amplitude, while the testing temperature change rate is setting as 5°C /min between -40°C to 40°C /min with liquid nitrogen.

In addition, the tensile measurements at low and high temperature have been performed by UTM (Zwick/Roell Z030, German). The printed samples and Poisson structure were stretched at a rate of 10 mm/min till the break took place, and the repetitive experiments have been measured at a stretching rate of 10 mm/min with a certain strain for different mixture of base materials

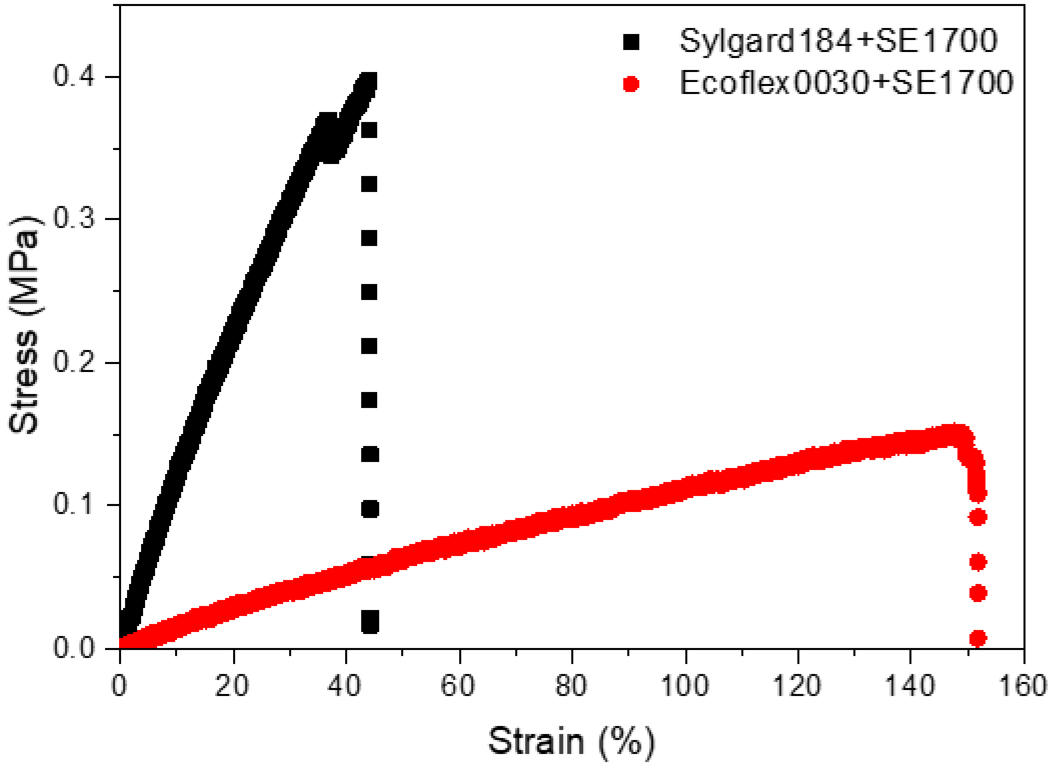

Figure S6 The load capacity versus the stretchable strain at low and high temperature for the Poisson structure with 60 vol% in LM.

Section 4. The electrical Properties of LM composites

The electrical resistance has been carried out by connecting copper line in the elastomer with LCR digital bridge (TH2827c, Tonghui, China). Meanwhile, the UTM has been applied to establish the stretching process.

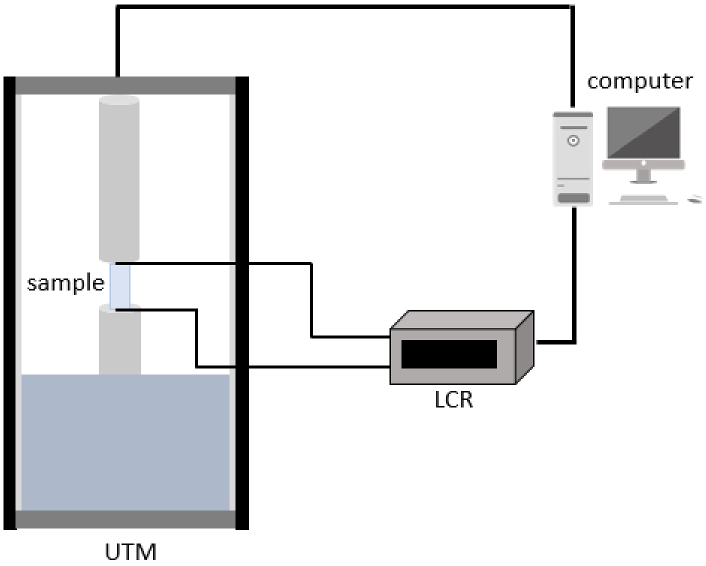

Figure S7. The Schematic diagram of real-time monitoring resistance measurements.

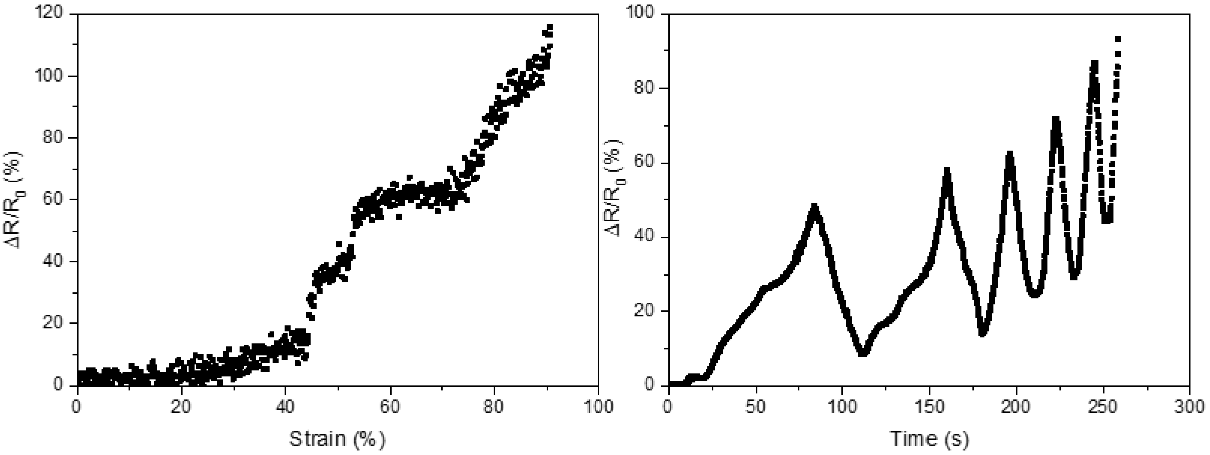

Figure S8. At high temperature (60 °C), the relative resistance of LM composites (that based on the mixture of Sylgard184 and SE 1700) changes with the stretchable strain, and b. the stretch repeatability over time.
